# Immunohistochemistry and Fluorescence In Situ Hybridization Can Inform the Differential Diagnosis of Low-Grade Noninvasive Urothelial Carcinoma with an Inverted Growth Pattern and Inverted Urothelial Papilloma

**DOI:** 10.1371/journal.pone.0133530

**Published:** 2015-07-24

**Authors:** Juan-Juan Sun, Yong Wu, Yong-Ming Lu, Hui-Zhi Zhang, Tao Wang, Xiao-Qun Yang, Meng-Hong Sun, Chao-Fu Wang

**Affiliations:** 1 Department of Pathology, Fudan University Shanghai Cancer Center, Shanghai, China; 2 Department of Oncology, Shanghai Medical College, Fudan University, Shanghai, China; 3 Institute of Pathology, Fudan University, Shanghai, China; Centro Nacional de Investigaciones Oncológicas (CNIO), SPAIN

## Abstract

Urothelial carcinoma (UC) comprises a heterogeneous group of epithelial neoplasms with diverse biological behaviors and variable clinical outcomes. Distinguishing UC histological subtypes has become increasingly important because prognoses and therapy can dramatically differ among subtypes. In clinical work, overlapping morphological findings between low-grade noninvasive UC (LGNUC), which exhibits an inverted growth pattern, and inverted urothelial papilloma (IUP) can make subclassification difficult. We propose a combination of immunohistochemistry (IHC) and molecular cytogenetics for subtyping these clinical entities. In our study, tissue microarray immunohistochemical profiles of Ki-67, p53, cytokeratin 20 (CK20) and cyclinD1 were assessed. Molecular genetic alterations such as the gain of chromosomes 3, 7 or 17 or the homozygous loss of 9p21 were also assessed for their usefulness in differentiating these conditions. Based on our analysis, Ki-67 and CK20 may be useful for the differential diagnosis of these two tumor types. Fluorescence in situ hybridization (FISH) can also provide important data in cases in which the malignant nature of an inverted urothelial neoplasm is unclear. LGNUC with an inverted growth pattern that is negative for both Ki-67 and CK20 can be positively detected using FISH.

## Introduction

Because of their diverse histological features, urothelial carcinomas (UCs) can be classified into a number of different morphological and architectural patterns [1–5]. These categories include an inverted or endophytic growth pattern. In practice, when only a small amount of biopsy material is available or when transurethral resections (for which tangential sectioning and thermal and crushing artifacts are important limitations) are examined, there is great overlap microscopically between low-grade noninvasive UC (LGNUC) with an inverted growth pattern and inverted urothelial papilloma (IUP), which is a rare benign tumor that accounts for approximately 1% to 2% of all urothelial neoplasms[6, 7]; thus, discriminating between these cases is problematic. However, whether to designate lesions as IUP or as LGNUC with an inverted growth pattern is important because their biological behaviors, subsequent treatments and prognoses differ.

We used various techniques, including immunohistochemistry (IHC) and fluorescence in situ hybridization (FISH) to determine the intrinsic differences between these two lesions and to correlate the findings with the need for a differential diagnosis.

## Materials and Methods

### Ethics statement

The study was approved by the ethics committee of Fudan University Shanghai Cancer Center. We obtained oral informed consent from the participants or their next of kin to use their tissues in this study. As the patients agreed to, their consent was recorded with their names. Additionally, the ethics committee of Fudan University Shanghai Cancer Center approved this consent procedure. Patient records/information were anonymized and de-identified prior to the analysis. All of the procedures were performed in accordance with the Declaration of Helsinki and relevant policies in China.

### Patient samples

The study comprised 38 specimens of LGNUC with an inverted growth pattern and 36 specimens of IUP, all of which were obtained from the Department of Pathology of Fudan University Shanghai Cancer Center. The tissues were fixed with buffered formalin (pH 7.0) and embedded in paraffin wax.

### Histological classification

The IUP diagnosis was based on the criteria set forth in the World Health Organization Classification of Tumors of the Urinary System and Male Genital Organs. A diagnosis of LGNUC with an inverted growth pattern was based on a number of histological features that have been described the literature[1, 8–10], including very thick columns or cords of neoplastic cells, irregularity in the width of these columns or cords, areas with unequivocal stromal invasion, cytologic atypia (nuclear pleomorphism, irregular nuclear contours, irregular chromatin distribution, and prominent nucleoli), and increased mitotic activity.

### Tissue microarray and IHC

The urothelial tissue microarray (TMA) was constructed with formalin-fixed, paraffin-embedded urothelial samples, and the areas of noninvasive UC or UP were identified according to the corresponding hematoxylin and eosin (H&E) stained slides. Two tissue core samples of low-grade UC with an inverted growth pattern and two IUP cores from the same case were arranged on a recipient paraffin block (1 mm core). IHC was performed using the Envision System with diaminobenzidine (DAB; Gene Tech, Shanghai Company Limited) according to the manufacturer’s protocol.

IHC was performed with the following antibodies: Ki-67 (clone MIB-1, DAKO Corp., Carpinteria, CA), p53 (prediluted antibody, DAKO Corp., Carpinteria, CA), CK20 (cloneKs20.8, prediluted antibody; DAKO Corp., Carpinteria, CA), and CyclinD1 (prediluted antibody, DAKO Corp., Carpinteria, CA). The extent of immunohistochemical staining was microscopically evaluated. The Ki-67 labeling was considered positive when moderate-to-strong staining was observed in greater than 1% of the tumor cells [11, 12]. The P53 and CycliD1 labeling were considered positive when moderate-to-strong staining was observed in greater than 10% of the tumor cells [13–15]. The CK20 labeling was considered positive when moderate-to-strong staining was observed in greater than 5% of the tumor cells [11, 12, 16].

### FISH

FISH analysis was performed as previously described [8]. Briefly, multiple 4-mm-thick unstained sections of each tumor were prepared from buffered formalin-fixed, paraffin-embedded tissue blocks. An H&E-stained slide from each case was examined by a pathologist to identify neoplastic tissue areas for FISH analysis. The slides were deparaffinized with 2 15-minute washes in xylene and 2 10-minute washes in absolute ethanol and were then air-dried in a fume hood. Next, the slides were treated with 0.1 mM citric acid (pH 6.0) (Zymed, South San Francisco, CA) at 95°C for 10 minutes, rinsed in distilled water for 3 minutes, and then washed in 2x standard saline citrate for 5 minutes. Digestion was performed by applying 0.4 mL of pepsin (5 mg/mL in 0.1 N HCl/0.9 NaCl) (Sigma, St Louis, MO) at 37°C for 40 minutes. The slides were rinsed with distilled water for 3 minutes, washed with 2x standard saline citrate for 5 minutes and air-dried. The α-satellite repeat (CEP) and locus-specific (LSI) probes were labeled with fluorophores. CEP3, CEP7, and CEP17 probes were labeled with Spectrum Red, Spectrum Green, and Spectrum Aqua. LSI p16 (9p21) was labeled with Spectrum Gold (Vysis, Downers Grove, America). The probes were diluted with tDenHyb2 (Insitus, Alburquerque, NM) at a ratio of 1:10. Five microliters of diluted probes were added to each slide under reduced-light conditions. Each slide was then covered with a 22mm × 22mm cover slip, and the edge was sealed with rubber cement. The slides were placed into an opaque plastic box that was wrapped with aluminum foil. The slides were denatured at 83°C for 12 minutes and hybridized at 37°C overnight. After hybridization, the slides were washed twice with prewarmed 0.1×SSC with 1.5 M urea at 45°C for 20 minutes each and were then washed with 2×SSC (saline sodium citrate) for 10 minutes and 2×SSC/0.1% NP40 for 10 minutes at 45°C. The slides were further washed with 2×SSC for 5 minutes at room temperature. The slides were counterstained with 10 μl 4',6-diamidino-2-phenylindole (DAPI; Vysis, Downers Grove, America) for 2 minutes and then covered with a 50×22 cover slip; the edges were then sealed with nail polish.

The stained slides were observed and documented using a Metasystem system (Belmont, MA) under a 100× oil objective. Positivity or negativity for specific chromosomal abnormalities was assessed in the tumor areas that exhibited an inverted growth pattern. The following filters were used: SP-100 for DAPI, FITC MF-101 for Spectrum Green, Gold 31003 for Spectrum Gold, Aqua 31036V2 for spectrum Aqua, and Texas Red Sp103 for Spectrum Red. Signals from each probe were counted from false color images in which the computer mapped each color channel into red, green, gold, and aqua. Five sequential focus stacks with 0.4-mm intervals were acquired and then integrated into a single image to reduce thickness-related artifacts.

For each case, 100 nuclei were counted. Each cell was simultaneously analyzed for the signals of chromosomes 3, 7, and 17 and that of 9p21. Twelve normal bladder specimens from patients who had no concurrent or prior history of UC or in situ carcinoma were used as negative controls, from which cutoff values were defined. Typically, 2 signals from each probe were measured in each single diploid cell.

The chromosomal gain or loss was defined based on the Gaussian model and was relative to normal controls. The cutoff values were set as the mean+3×SD (standard deviation) of the number of disomic cells in control individuals. The mean+3×SD represents a specificity of 99.9%. Any tumor case with a score beyond the cutoff value was considered to have either a gain or a loss of the designated chromosomes. Cases that exhibited a gain of 2 or more of chromosomes 3, 7, and 17 or a loss of 9p21 were defined as positive.

### Statistical analysis

The data were analyzed using the SPSS 18.0 software package (SPSS, Chicago, Illinois, USA). A blinded comparison of the sensitivity and specificity of histological analysis compared with that of immunocytochemistry analysis was performed, with independent researchers analyzing the H&E sections and ICC stains. Fisher's exact test was performed to compare the IHC and FISH results between IUP and LGNUC with an inverted growth pattern. In this study, disease-free survival (DFS) was calculated using the Kaplan–Meier estimator implemented in the GraphPad Prism 5 software package (GraphPad Software Inc., San Diego, CA, USA), and statistical significance was defined as P<0.05.

## Results

### 1. Morphological analysis

**Fig 1:**
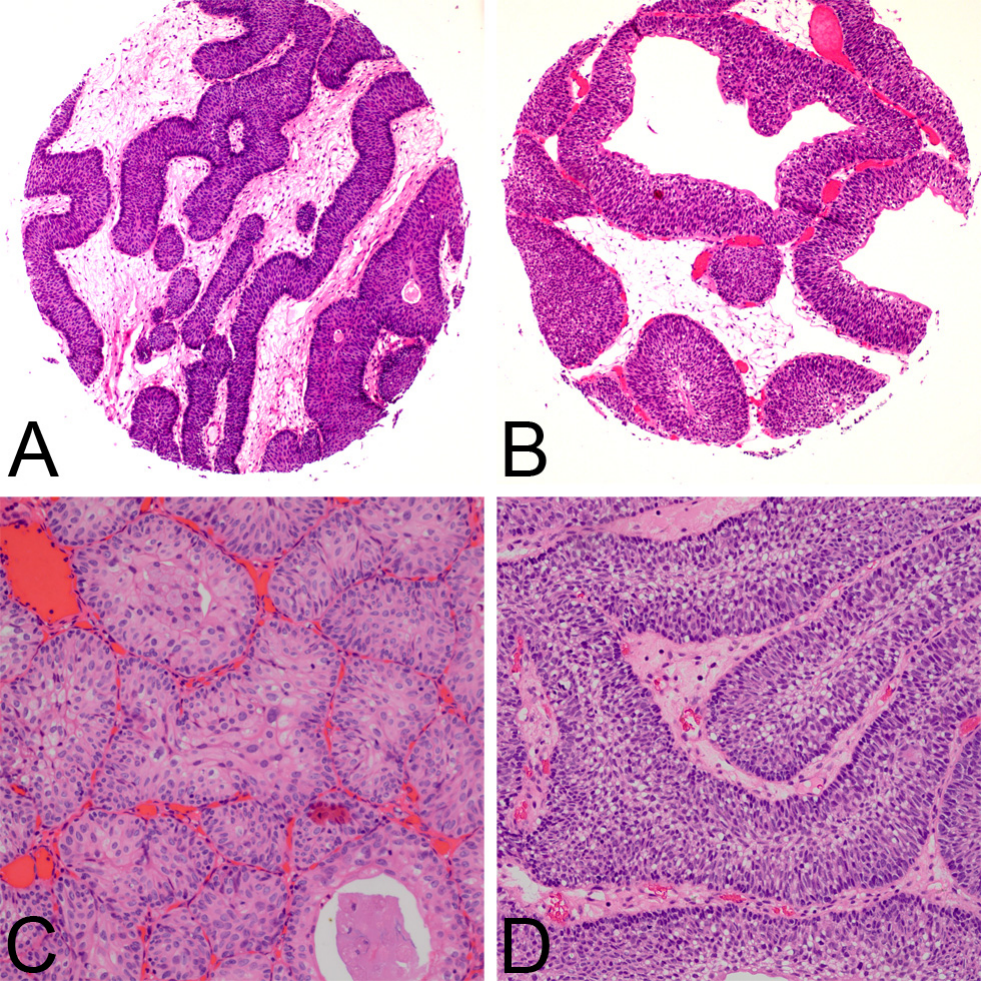
Clinicopathological features of inverted papilloma. IUPs were mainly comprised anastomosing cords, which were thin and congruous in width **(A)**, or nests **(C)**. Cysts were occasionally observed in the interiors of the nests **(B)**. Peripheral palisading nuclei were observed **(D)**.

**Fig 2:**
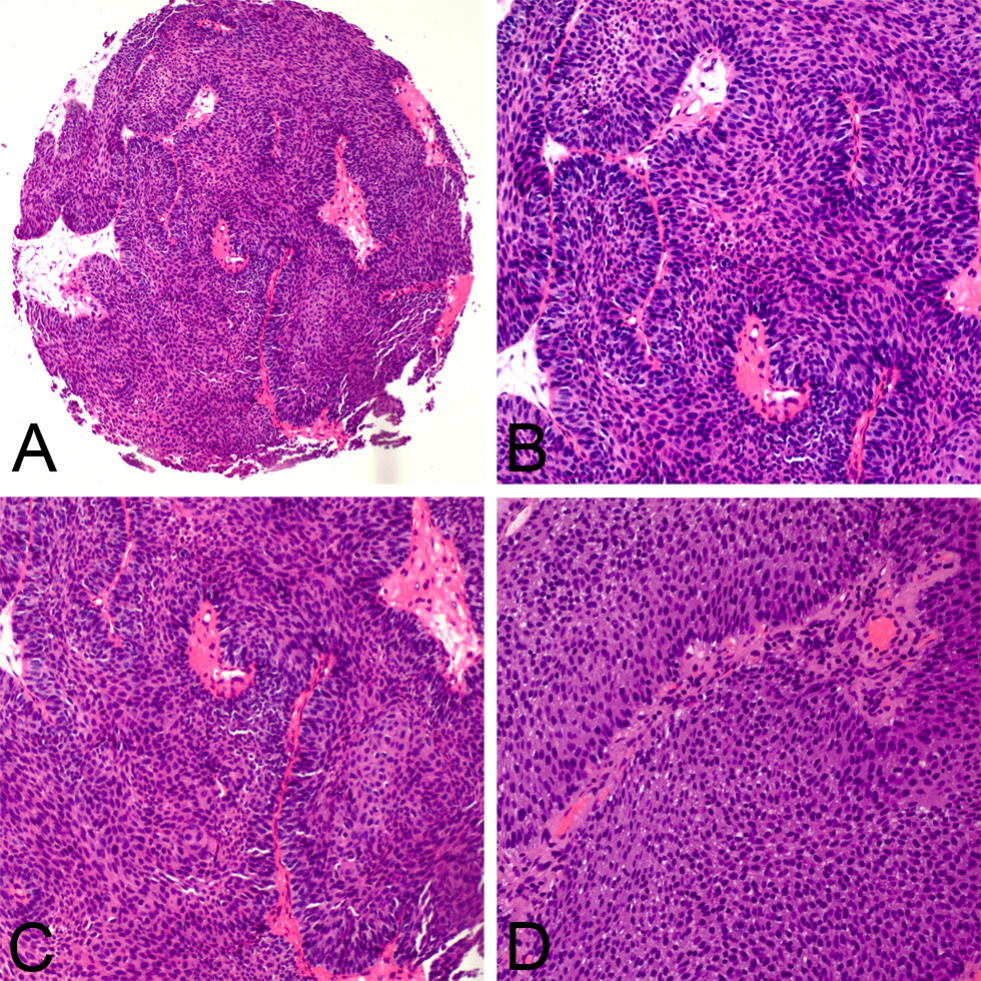
Clinicopathological features of low-grade noninvasive UC with an inverted growth pattern. The cords of LGNUC with an inverted growth pattern were usually thick and irregular (A), and definite fibrovascular cores were observed (B). Nests were usually large and inordinate (C), and mitotic figures, which were common in the superficial or middle layers, were easy to detect (D).

The morphological comparison of IUP and LGNUC with an inverted growth pattern is summarized in Table 1. All 36 inverted papillomas mainly comprised anastomosing cords, which were thin and congruous in width, or nests, which were small and regular (Fig 1). Cysts were occasionally observed in the interiors of the nests. Of the 36 inverted papillomas, peripheral palisading nuclei were observed in 31 cases (86.1%), and streaming in the inferior of cords or nests was observed in 28 cases (77.8%). Mitotic figures were seldom observed; they were only observed in the basal layer. Less than 1/10 high-power field (HPF) mitotic figures were found in all of the inverted papilloma cases in this study. The cords of LGNUC with an inverted growth pattern were usually thick and irregular, and the nests were usually large and inordinate (Fig 2). Definite fibrovascular cores were observed in 17 of 38 cases (44.7%). Mitotic figures, which were common in superficial or middle layers, were easy to detect in 20 of the 38 cases (52.6%) that exhibited >1/10 HPF in this study.

**Table 1:** The morphological comparison of IUP and LGNUC with an inverted growth pattern.

	IUP	LGNUC with inverted growth pattern
**Endophytic cords of urothelial cells**	24/36 (63.2%), thin and congruous in width, cysts could be seen	30/38(78.9%), thick and irregular
**Endophytic nests of urothelial cells**	12/36 (36.8%), small and regular	8/39(21.1%), big and inordinate
**Peripheral palisading**	31/36 (86.1%)	19/38 (50%)
**Streaming in the inferior of cords or nests**	28/36 (77.8%)	15/38 (44.7%)
**Fibrovascular cores**	0	17/38 (44.7%)
**Mitotic activity**		
**<1/10 HPF**	36/36 (100%)	18/38 (47.4%)
**≥1/10 HPF**	0 (0%)	20/38 (52.6%)

### 2. IHC results (Table 2)

**Fig 3:**
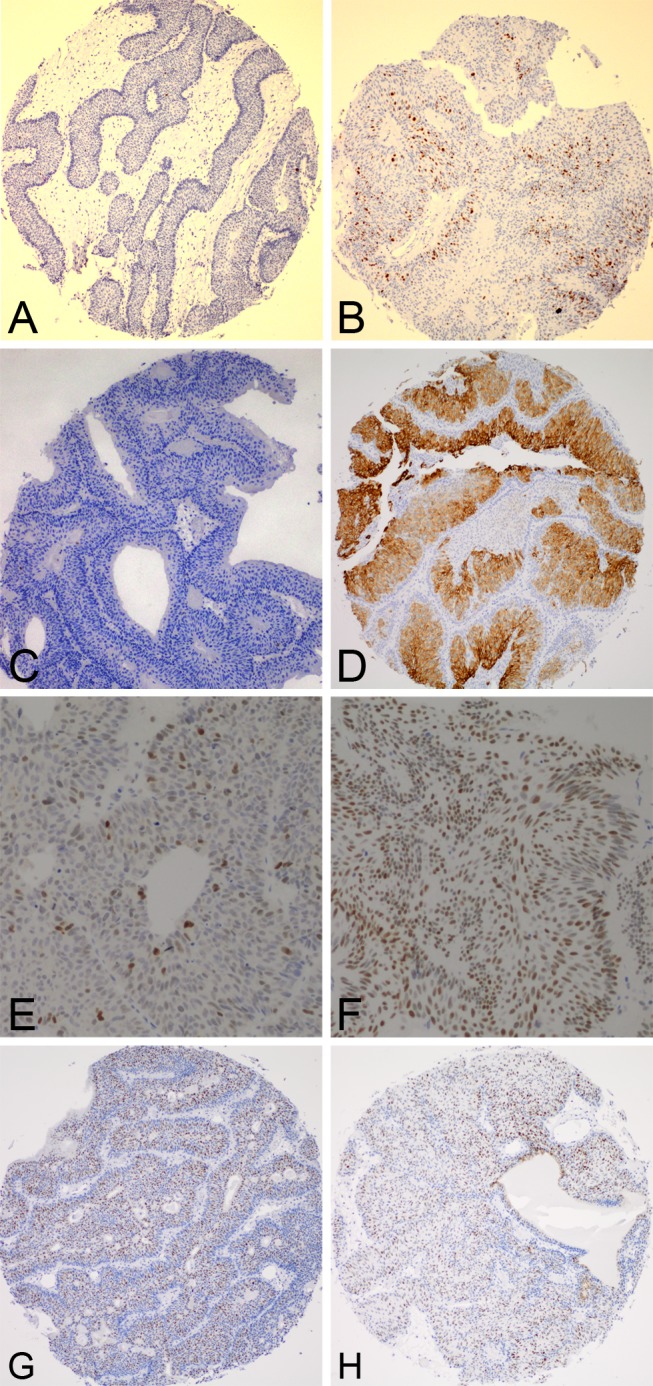
IHC results for the two tumor types. Ki-67 was negative for IUP **(A)** but positive for LGNUC with an inverted growth pattern **(B)**. CK20 was negative for IUP **(C)** but positive for LGNUC with an inverted growth pattern **(D)**. IUP **(E)** and LGNUC with an inverted growth pattern **(F)** were both positive for P53. IUP **(G)** and LGNUC with an inverted growth pattern **(H)** were both positive for CyclinD1.

1. Ki-67 expression was significantly lower in IUP (Fig 3A), for which only 1 of 36 cases (2.8%) was positive, than in LGNUC with an inverted growth pattern (Fig 3B), for which 18 of 38 cases (47.4%) were positive (*P*<0.001). CK20 expression was significantly lower in IUP (Fig 3C), for which 2 of 36 cases (5.6%) were positive, than in LGNUC with an inverted growth pattern (Fig 3D), for which 14 of 38 cases (36.8%) were positive (*P* = 0.001). P53 expression was lower in IUP (Fig 3E), for which 10 of 36 cases (27.8%) were positive, than in LGNUC with an inverted growth pattern (Fig 3F), for which 16 of 38 cases (42.1%) were positive, but the difference between them was not statistically significant (*P* = 0.230). CyclinD1 expression was slightly lower in IUP (Fig 3G), for which 25 of 36 cases (69.4%) were positive, than in LGNUC with an inverted growth pattern (Fig 3H), for which 28 of 38 cases (73.7%) were positive, but the difference between them was not statistically significant (*P* = 0.798).

**Table 2:** Comparison of immunohistochemical features of IUP and LGNUC with an inverted growth pattern.

Types	Cases	Ki-67		CK20		P53		CyclinD1	
		IR	P	IR	P	IR	P	IR	P
**IP**	36	1	0.000	2	0	10	0.001	25	**0.798**
**LGNUC**	38	18		14		16		28	

IR, Immunoreactivity.

2) After combining Ki-67 with CK20 for the differential diagnosis (Table 3), 3 of 36 cases of IUP (8.3%) had one positive marker, and 13 of 38 cases of LGNUC with an inverted growth pattern (34.2%) were not positive for either of these two markers.

**Table 3:** The distribution of Ki-67 and CK20 in IUP and LGNUC with an inverted growth pattern.

Types	All negative	At least one positive	All positive	Cases
**IUP**	33 (91.7%)	3 (8.3%)	0	36
**LGNUC with an inverted growth**	13 (34.2%)	25 (65.8%)	7(18.4%)	38

### 3. FISH results

**Fig 4:**
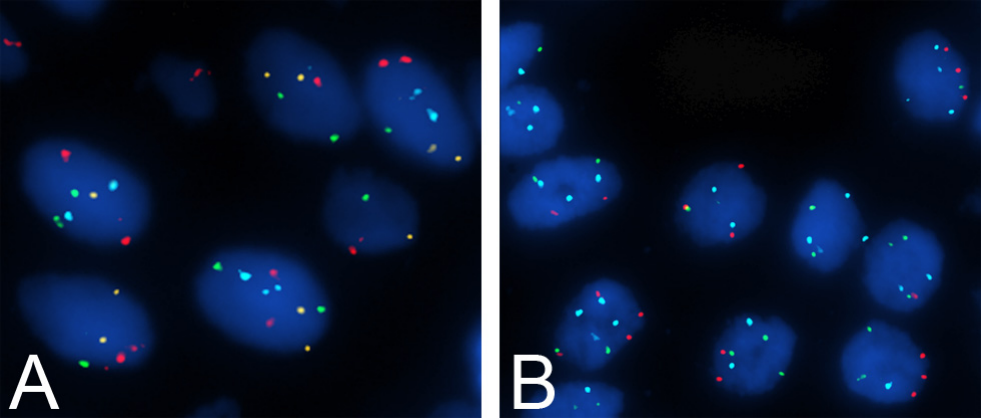
FISH results for the two tumor types. The cases that exhibited a gain of at least 2 of chromosomes 3 **(B)**, 7 **(B)** and 17 **(A, B)** or a homozygous loss of 9p21 **(B)** were defined as UroVysion FISH positive.

Cases that exhibited a gain of at least 2 of chromosomes 3, 7 and 17 or a homozygous loss of 9p21 (Fig 4A and 4B) were defined as UroVysion FISH positive, and the normal values are listed in Table 4. Positive results were obtained in 24 of the 38 cases (63.2%) of LGNUC with an inverted growth pattern, whereas negative results were obtained in all of the IUP cases; 9 of the 13 (69.2%) cases that were negative in terms of both Ki-67 and CK20 were positive in terms of UroVysion FISH. We compared the sensitivity and specificity of Ki-67, CK20, and FISH between IUP and LGNUC with an inverted growth pattern (Table 5). When Ki-67 and CK20 were combined with UroVysion FISH, the sensitivity and specificity were 89.5% and 97%, respectively.

**Table 4:** The normal values of chromosomes 3, 7 and 17 or of 9p21.

	3(%)	7(%)	17(%)	9p21(%)
**Mean value**	2.76	1.18	0.97	9.27
**Standard deviation**	2.39	1.36	1.47	4.02
**Reference range**	0~9.92	0~5.25	0~5.37	0~21.32

**Table 5:** Comparison of the sensitivity and specificity of Ki-67, CK20, FISH in IUP and LGNUC with an inverted growth pattern.

	sensitivity	specificity
**Ki-67**	18/38(47.4%)	35/36(97.2%)
**CK20**	14/38(36.8%)	34/36(94.4%)
**FISH**	24/38(63.2%)	33/33(100%)
**Ki-67 combining with CK20**	25/38(65.8%)	33/36(91.7%)
**The combination of the three**	34/38(89.5%)	33/33(100%)

### 4. Survival analysis

**Fig 5:**
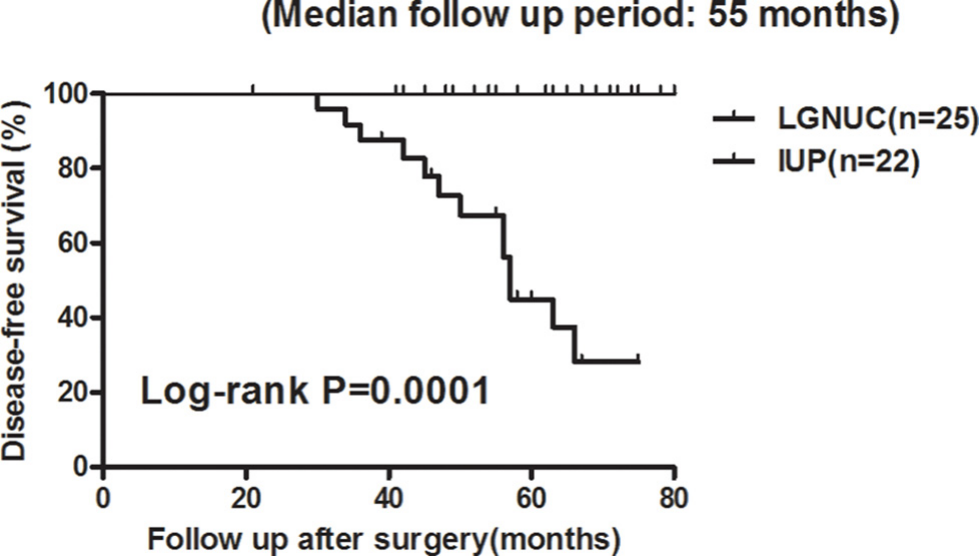
Comparison between the DFS of the IUP group and that of the LGNUC with an inverted growth pattern group.

LGNUC with an inverted growth pattern and IUP are associated with different treatment approaches and prognoses. These conclusions are also verified in our study. Follow-up data were available for 47 patients, including 22 IUP and 25 LGNUC with an inverted growth pattern. The Kaplan–Meier estimate indicated that the groups of patients with these two tumors had significantly different DFS values (P = 0.0001, Fig 5).

## Discussion

IUP is an uncommon urinary tract tumor that is usually regarded as benign [17–20]. Of a sample of 1829 urothelial tract tumors, only 2.2% were IUPs [21]. Since the first description of IUP by Potts and Hirst in 1963 [22], more than 1000 cases have been reported. Witjes et al [19] concluded that IUP was not a risk factor for transitional cell carcinoma (TCC) of the urinary tract and that frequent and long-term follow-up was not required if the histological diagnosis was definitive. Christopher Cheng et al [20] reviewed urinary bladder cases and determined that cystoscopic surveillance may not be necessary in histologically proven solitary bladders with no associated TCC. Although IUP has been described to undergo malignant transformation to some extent [21, 23–28], most studies have classified IUP as a benign neoplasm.

However, in practice, distinguishing between these two diseases based on morphology alone is sometimes difficult. Because of this difficulty, Risio et al[29] stated that some IUP cases are possibly diagnosed as LGNUC with an inverted growth pattern and that the actual incidence of IUP might be higher than indicated by the reported figures. During the present study, we examined a series of 38 cases of LGNUC with an inverted growth pattern and 36 cases of IUP using IHC and FISH. Timothy D. Jones et al [8] demonstrated that these ancillary techniques can validate the diagnosis of these diseases, but their sample size was not sufficiently large to provide convincing results. To the best of our knowledge, the present study represents the largest series used to differentiate these clinical entities that has been presented to date.

The clinical features of LGNUC with an inverted growth pattern and IUP are not specific, and these conditions maybe share similar histological appearances. However, histological examination may still hold the key for diagnostic accuracy, and most cases can be diagnosed on morphological grounds alone. Amin et al [30] analyzed 18 UCs with a prominent endophytic growth component and emphasized the problems associated with the histological assessment of invasion and the different patterns of invasion observed in these tumors. They then established a number of morphological criteria that can be used to distinguish IUPs from UCs with a prominent endophytic growth pattern These same criterion were used and validated in the current study, as detailed above, but when they share very similar morphological features, these criterion maybe ineffective clinically. Henderson et al [31] defined six diagnosis criteria for inverted papilloma. Moreover, Broussard et al [32] subdivided the IUP cases with atypia into five groups (although the classification was not widely accepted). As described in the literature, the typical appearance of an IUP is that of a polyploid lesion covered in a flattened but otherwise normal-looking layer of transitional cell epithelium[1, 3, 19]. Beneath this surface, numerous communicating and interdigitating epithelial strands lie within a characteristic loose connective tissue stroma. Although slight atypia may be observed, the tumor cells have a monomorphic and often basaloid appearance, with a tendency toward palisading at the periphery and spindling in the center of the epithelial strands, and mitotic figures are rare or absent[7, 33]. In contrast, the surface of an inverted papilloma-like UC is sometimes exophytic and variable, and the ramifying cords and trabeculae have irregular widths, which are not characteristic features of inverted papillomas. Cell atypia, nuclear pleomorphism, chromatin distribution irregularities, and a faster mitotic rate are the prominent features in the diagnosis of TCCs. However, the typical appearance above does not always occur for low-grade noninvasive UC with an inverted growth pattern, thereby making diagnosis based only on microscopic findings difficult. In our series, the selected tumors were all LGNUC with an inverted growth pattern, which enabled a better comparison between these two tumors.

Numerous reports have investigated the immunohistochemical reactivity of UC and IUP. Urakami et a1 [23] investigated p53 and proliferating cell nuclear antigen immunoreactivity in addition to intense Feulgen staining in IUPs and concluded that these lesions might have high proliferative activity and that IUPs with a high immunoreactivity for p53 may be susceptible to malignant transformation. Similar findings were also reported by Kunimi et al [34]. Deoxyribonucleic acid (DNA) analysis of 8 patients revealed diploid histograms in all except 1 patient, but the IUPs had relatively higher proliferative activity (the average percentage of the S phase in the tumor DNA histograms was 9.78%, whereas in normal DNA, it was 4.87). One patient exhibited an aneuploid DNA pattern (index 1.26) and subsequently had a bladder carcinoma. Valero Puerta et al[35] determined the proliferative activity using Ki-67 antigen expression in 12 patients with IUPs. They also concluded that a higher proliferative activity was generally associated with poorer outcomes. Generally, TCCs and non-advanced, high-grade TCCs frequently express biomarkers such as Ki-67, p53, and CK20, although IUPs rarely exhibited immunoreactivity for these biomarkers. CyclinD1 expression varies, but higher expression has been reported in superficial low-grade TCCs and not in advanced high-grade TCCs [9, 23, 36]. These biological characteristics of IUPs or inverted papilloma-like TCCs have not yet been sufficiently evaluated. However, one report demonstrated a single case of TCC with inverted papilloma-like growth with high p53 immunoreactivity, similar to the current study[23]. In the present study, we chose to use Ki-67, P53, CK20, and cyclinD1 as biomarkers, and cases of LGNUC with an inverted growth pattern expressed one or more of these proteins similarly to their non-inverted counterparts. In contrast, IUP specimens exhibited sparse immunoreactivity.

A variety of molecular genetic alterations in urothelial tumors have been reported and have been detected using many techniques, including loss of heterozygosity (LOH) analysis and FISH [37–43]. Most low-grade, noninvasive papillary UCs and some high-grade lesions are characterized by the loss of chromosome 9[44–46]. In contrast, p53 gene mutations and the loss of chromosome 17p are primarily observed in high-grade, invasive UCs [44, 47, 48]. A model of urothelial carcinogenesis in which the loss of chromosome 9 is the initial step that leads to a low-grade papillary lesion, with invasion and progression characterized by later p53 gene mutations, has been proposed. In in situ UCs, p53 gene mutations occur earlier [44, 49]. Additionally, IUPs do not exhibit the genetic-alteration-based molecular features that UCs often exhibit. Our study employed UroVysion, which is a multitarget FISH assay, to detect aneuploidy in chromosomes 3, 7, and 17 and loss of the 9p21 locus using a fluorescence microscope. This test has been approved by the FDA for use in urine cytology both for monitoring for recurrence in patients with a history of bladder cancer and for detecting new malignancies in patients with hematuria. In this study, we applied the principles of UroVysion FISH to formalin-fixed, paraffin-embedded tissues using statistically derived cutoff values, as discussed above. We observed that 24 of 38 cases (63.2%) of LGNUC with an inverted growth pattern were positive, while all cases of IUP were negative; 9 of 13 (69.2%) cases that were negative for both Ki-67 and CK20 were determined to be positive by UroVysion FISH. When Ki-67 and CK20 were combined with UroVysion FISH, the sensitivity and specificity were 89.5% and 97%, respectively. Thus, IUP likely arises from pathogenetic mechanisms that differ from those that produce UC. Additionally, UroVysion FISH may be an important approach in cases in which the malignant nature of an inverted urothelial neoplasm is unclear.

Although not included in the current study, other techniques or biomarkers such as flow cytometry or proliferating cell nuclear antigen (PCNA) have been used to distinguish these two tumors. Sung and colleagues [50] examined a series of 39 IUPs using LOH analysis and demonstrated a very low incidence of LOH at genetic loci that are frequently lost in both UCs and papillary urothelial neoplasms of low malignant potential. In fact, the incidence of LOH in these IUPs was similar to that previously reported for normal urothelium. Our results are consistent with those of Sung et al in the sense that none of the IUPs in this study exhibited molecular features of urothelial cancer via the UroVysion FISH analysis. Jun Cheon et al[51] studied the indicators of cellular proliferative activity in seven bladder IUPs, including two cases of malignant IUP. In that study, the PCNA expression rates in two cases of malignant inverted papilloma were higher than in benign IUPs. The mean numbers of argyrophilic nucleolar organizer regions (AgNORs) per nucleus in malignant inverted papillomas were much higher than in benign inverted papillomas. The c-erbB-2 oncoprotein was expressed only in malignant inverted papillomas. Thus, the above phenomenon may explain the favorable prognosis and benign clinical course of inverted papilloma.

In summary, our study is the largest series to date to differentiate between these two tumors. Because LGNUC with an inverted growth pattern and IUP sometimes have an analogous histological appearance, ancillary techniques such as IHC and FISH can help in the differential diagnosis of LGNUC with an inverted growth pattern and IUP.

## References

[1] Eble JN, Sauter G, Epstein JI, Sesterhenn IA. World Health Organization Classification of Tumors:Pathology and Genetics of Tumours of the Urinary System and Male Genital Organs. Lyon: IARC Press.

[2] Perez-Montiel DÍSS. Upper urinary tract carcinomas: histological types and unusual morphological variants. Diagnostic Histopathology. 2008;14(1):48–54.

[3] Humphrey PA. Urinary bladder pathology 2004: An update. Annals of Diagnostic Pathology. 2004;8(6):380–9. 15614746 10.1053/j.anndiagpath.2004.08.012

[4] Drew PA, Furman J, Civantos F, Murphy WM. The nested variant of transitional cell carcinoma: an aggressive neoplasm with innocuous histology. Modern pathology: an official journal of the United States and Canadian Academy of Pathology, Inc. 1996;9(10):989–94. 8902836

[5] Wasco MJ, Daignault S, Bradley D, Shah RB. Nested variant of urothelial carcinoma: a clinicopathologic and immunohistochemical study of 30 pure and mixed cases. Hum Pathol. 2010;41(2):163–71. 10.1016/j.humpath.2009.07.015 19800100

[6] Mattelaer J, Leonard A, Goddeeris P, D'Hoedt M, Van Kerrebroeck P. Inverted papilloma of bladder: clinical significance. Urology. 1988;32(3):192–7. 3413911 10.1016/0090-4295(88)90383-4

[7] Cameron KM, Lupton CH. Inverted papilloma of the lower urinary tract. Br J Urol. 1976;48(7):567–77. 1016830 10.1111/j.1464-410x.1976.tb06703.x

[8] Jones TD, Zhang S, Lopez-Beltran A, Eble JN, Sung MT, MacLennan GT, et al Urothelial carcinoma with an inverted growth pattern can be distinguished from inverted papilloma by fluorescence in situ hybridization, immunohistochemistry, and morphologic analysis. The American journal of surgical pathology. 2007;31(12):1861–7. Epub 2007/11/29. 10.1097/PAS.0b013e318060cb9d . 18043040

[9] Sudo T, Irie A, Ishii D, Satoh E, Mitomi H, Baba S. Histopathologic and biologic characteristics of a transitional cell carcinoma with inverted papilloma-like endophytic growth pattern. Urology. 2003;61(4):837. 10.1016/s0090-4295(02)02521-9 12670580

[10] Spevack L, Herschorn S, Srigley J. Inverted papilloma of the upper urinary tract. J Urol. 1995;153(4):1202–4. 7869499

[11] Ye YK, Bi XC, He HC, Han ZD, Dai QS, Liang YX, et al CK20 and Ki-67 as significant prognostic factors in human bladder carcinoma. Clinical and experimental medicine. 2010;10(3):153–8. Epub 2010/01/14. 10.1007/s10238-009-0088-3 . 20069333

[12] Mallofre C, Castillo M, Morente V, Sole M. Immunohistochemical expression of CK20, p53, and Ki-67 as objective markers of urothelial dysplasia. Modern pathology: an official journal of the United States and Canadian Academy of Pathology, Inc. 2003;16(3):187–91. Epub 2003/03/18. 10.1097/01.mp.0000056628.38714.5d . 12640096

[13] Lee K, Jung ES, Choi YJ, Lee KY, Lee A. Expression of pRb, p53, p16 and cyclin D1 and their clinical implications in urothelial carcinoma. Journal of Korean medical science. 2010;25(10):1449–55. Epub 2010/10/05. 10.3346/jkms.2010.25.10.1449 ; PubMed Central PMCID: PMCPmc2946654. 20890425 PMC2946654

[14] Shariat SF, Bolenz C, Karakiewicz PI, Fradet Y, Ashfaq R, Bastian PJ, et al p53 expression in patients with advanced urothelial cancer of the urinary bladder. BJU Int. 2010;105(4):489–95. Epub 2009/08/08. 10.1111/j.1464-410X.2009.08742.x . 19659466

[15] Shariat SF, Ashfaq R, Sagalowsky AI, Lotan Y. Correlation of cyclin D1 and E1 expression with bladder cancer presence, invasion, progression, and metastasis. Hum Pathol. 2006;37(12):1568–76. Epub 2006/09/05. 10.1016/j.humpath.2006.05.017 . 16949911

[16] Harnden P, Mahmood N, Southgate J. Expression of cytokeratin 20 redefines urothelial papillomas of the bladder. Lancet. 1999;353(9157):974–7. Epub 1999/08/25. 10.1016/s0140-6736(98)05383-5 . 10459907

[17] Rozanski TA. Inverted papilloma: an unusual recurrent, multiple and multifocal lesion. J Urol. 1996;155(4):1391 8632588 10.1016/s0022-5347(01)66280-3

[18] Sung M-T, MacLennan GT, Lopez-Beltran A, Montironi R, Cheng L. Natural history of urothelial inverted papilloma. Cancer. 2006;107(11):2622–7. 17078053 10.1002/cncr.22311

[19] Witjes JA, van Balken MR, van de Kaa CA. THE PROGNOSTIC VALUE OF A PRIMARY INVERTED PAPILLOMA OF THE URINARY TRACT. The Journal of Urology. 1997:1500–5. 9302151

[20] Ho H, Chen YD, Tan PH, Wang M, Lau WKO, Cheng C. Inverted papilloma of urinary bladder: Is long-term cystoscopic surveillance needed? A single center¡¯s experience. Urology. 2006;68(2):333–6. 16904447 10.1016/j.urology.2006.03.014

[21] Kunze E, Schauer A, Schmitt M. Histology and histogenesis of two different types of inverted urothelial papillomas. Cancer. 1983;51(2):348–58. 6821821 10.1002/1097-0142(19830115)51:2<348::aid-cncr2820510231>3.0.co;2-o

[22] Potts IF, Hirst E. INVERTED PAPILLOMA OF THE BLADDER. J Urol. 1963;90:175–9. 14044188 10.1016/S0022-5347(17)64384-2

[23] Urakami S, Igawa M, Shirakawa H, Shiina H, Ishibe T. Biological characteristics of inverted papilloma of the urinary bladder. Br J Urol. 1996;77(1):55–60. 8653317 10.1046/j.1464-410x.1996.08209.x

[24] Anderstrom C, Johansson S, Pettersson S. Inverted papilloma of the urinary tract. J Urol. 1982;127(6):1132–4. 7087020 10.1016/s0022-5347(17)54266-4

[25] DeMeester LJ, Farrow GM, Utz DC. Inverted papillomas of the urinary bladder. Cancer. 1975;36(2):505–13. 50875 10.1002/1097-0142(197508)36:2<505::aid-cncr2820360229>3.0.co;2-l

[26] Picozzi S, Casellato S, Bozzini G, Ratti D, Macchi A, Rubino B, et al Inverted papilloma of the bladder: A review and an analysis of the recent literature of 365 patients. Urologic Oncology: Seminars and Original Investigations. 2013;31(8):1584–90. 10.1016/j.urolonc.2012.03.009 22520573

[27] Kimura G, Tsuboi N, Nakajima H, Yoshida K, Masugi Y, Akimoto M. Inverted papilloma of the ureter with malignant transformation: a case report and review of the literature. The importance of the recognition of the inverted papillary tumor of the ureter. Urol Int. 1987;42(1):30–6. 3296385 10.1159/000281846

[28] Palvio DH. Inverted papillomas of the urinary tract. A case of multiple, recurring inverted papillomas of the renal pelvis, ureter and bladder associated with malignant change. Scand J Urol Nephrol. 1985;19(4):299–302. 4089556 10.3109/00365598509180275

[29] Risio M, Coverlizza S, Lasaponara F, Vercesi E, Giaccone G. Inverted urothelial papilloma: a lesion with malignant potential. Eur Urol. 1988;14(4):333–8. 3169075 10.1159/000472973

[30] Amin MB, Gomez JA, Young RH. Urothelial transitional cell carcinoma with endophytic growth patterns: a discussion of patterns of invasion and problems associated with assessment of invasion in 18 cases. The American journal of surgical pathology. 1997;21(9):1057–68. 9298882 10.1097/00000478-199709000-00010

[31] Henderson DW, Allen PW, Bourne AJ. Inverted urinary papilloma: report of five cases and review of the literature. Virchows Arch A Pathol Anat Histol. 1975;366(3):177–86. 805489 10.1007/BF00427408

[32] Broussard JN, Tan PH, Epstein JI. Atypia in inverted urothelial papillomas: Pathology and prognostic significance. Human Pathology. 2004;35(12):1499–504. 15619209 10.1016/j.humpath.2004.09.010

[33] Romanelli R. Inverted urothelial papilloma. Report of five cases and review of the literature. Pathologica. 1986;78(1053):89–97. 3822559

[34] Kunimi K, Uchibayashi T, Hasegawa T, Lee SW, Ohkawa M. Nuclear deoxyribonucleic acid content in inverted papilloma of the urothelium. Eur Urol. 1994;26(2):149–52. 7957471 10.1159/000475366

[35] Valero PJA, Redondo ME, Jiemenez GC, Gomez MML, Martinez DLRSI. Inverted transitional-cell papilloma: the expression of the Ki-67 nuclear antigen as a prognostic factor. Arch Esp Urol. 1995;48(9):887–92. 8554393

[36] Sgambato A, Migaldi M, Faraglia B, De Aloysio G, Ferrari P, Ardito R, et al Cyclin D1 expression in papillary superficial bladder cancer: its association with other cell cycle-associated proteins, cell proliferation and clinical outcome. International journal of cancer Journal international du cancer. 2002;97(5):671–8. 11807796 10.1002/ijc.10055

[37] Cheng L, Gu J, Ulbright TM, MacLennan GT, Sweeney CJ, Zhang S, et al Precise microdissection of human bladder carcinomas reveals divergent tumor subclones in the same tumor. Cancer. 2002;94(1):104–10. 11815965 10.1002/cncr.10151

[38] Cheng L, MacLennan GT, Zhang S, Wang M, Pan CX, Koch MO. Laser capture microdissection analysis reveals frequent allelic losses in papillary urothelial neoplasm of low malignant potential of the urinary bladder. Cancer. 2004;101(1):183–8. 15222005 10.1002/cncr.20343

[39] Cheng L, Jones TD, McCarthy RP, Eble JN, Wang M, MacLennan GT, et al Molecular genetic evidence for a common clonal origin of urinary bladder small cell carcinoma and coexisting urothelial carcinoma. The American journal of pathology. 2005;166(5):1533–9. 15855652 10.1016/S0002-9440(10)62369-3 PMC1606405

[40] Jones TD, Carr MD, Eble JN, Wang M, Lopez-Beltran A, Cheng L. Clonal origin of lymph node metastases in bladder carcinoma. Cancer. 2005;104(9):1901–10. 16196038 10.1002/cncr.21466

[41] Cordon-Cardo C. Molecular alterations associated with bladder cancer initiation and progression. Scandinavian Journal of Urology and Nephrology. 2008;42(s218):154–65. 18815930 10.1080/03008880802291915

[42] Jones TD, Wang M, Eble JN, MacLennan GT, Lopez-Beltran A, Zhang S, et al Molecular evidence supporting field effect in urothelial carcinogenesis. Clinical cancer research: an official journal of the American Association for Cancer Research. 2005;11(18):6512–9. 10.1158/1078-0432.CCR-05-0891 16166427

[43] Sung MT, Wang M, MacLennan GT, Eble JN, Tan PH, Lopez-Beltran A, et al Histogenesis of sarcomatoid urothelial carcinoma of the urinary bladder: evidence for a common clonal origin with divergent differentiation. J Pathol. 2007;211(4):420–30. 17236170 10.1002/path.2129

[44] Spruck CHR, Ohneseit PF, Gonzalez-Zulueta M, Esrig D, Miyao N, Tsai YC, et al Two molecular pathways to transitional cell carcinoma of the bladder. Cancer Res. 1994;54(3):784–8. 8306342

[45] Knowles MA, Elder PA, Williamson M, Cairns JP, Shaw ME, Law MG. Allelotype of human bladder cancer. Cancer Res. 1994;54(2):531–8. 8275491

[46] Ruppert JM, Tokino K, Sidransky D. Evidence for two bladder cancer suppressor loci on human chromosome 9. Cancer Res. 1993;53(21):5093–5. 8221642

[47] Rosin MP, Cairns P, Epstein JI, Schoenberg MP, Sidransky D. Partial allelotype of carcinoma in situ of the human bladder. Cancer Res. 1995;55(22):5213–6. 7585577

[48] Esrig D, Spruck CHR, Nichols PW, Chaiwun B, Steven K, Groshen S, et al p53 nuclear protein accumulation correlates with mutations in the p53 gene, tumor grade, and stage in bladder cancer. The American journal of pathology. 1993;143(5):1389–97. 7901994 PMC1887166

[49] Jones PA, Droller MJ. Pathways of development and progression in bladder cancer: new correlations between clinical observations and molecular mechanisms. Semin Urol. 1993;11(4):177–92. 8290824

[50] Sung MT, Eble JN, Wang M, Tan PH, Lopez-Beltran A, Cheng L. Inverted papilloma of the urinary bladder: a molecular genetic appraisal. Modern pathology: an official journal of the United States and Canadian Academy of Pathology, Inc. 2006;19(10):1289–94. 10.1038/modpathol.3800667 16862073

[51] Cheon J, Kim HK, Kim JJ, Yoon DK, Koh SK, Kim IS. Malignant inverted papilloma of the urinary bladder: the histopathological aspect of malignant potential of inverted papilloma. Journal of Korean medical science. 1995;10(2):103–10. 7576290 10.3346/jkms.1995.10.2.103 PMC3054137

